# NRF2 and Thioredoxin Reductase 1 as Modulators of Interactions between Zinc and Selenium

**DOI:** 10.3390/antiox13101211

**Published:** 2024-10-08

**Authors:** Alina Löser, Maria Schwarz, Anna Patricia Kipp

**Affiliations:** 1 Department of Nutritional Physiology, Institute of Nutritional Sciences, Friedrich Schiller University Jena, 07743 Jena, Germany; alina.loeser@uni-jena.de (A.L.); schwarz.maria@uni-jena.de (M.S.); 2 TraceAge-DFG Research Unit on Interactions of Essential Trace Elements in Healthy and Diseased Elderly, Potsdam-Berlin-Jena-Wuppertal, 14558 Nuthetal, Germany

**Keywords:** zinc, selenium, NRF2, thioredoxin reductase, selenium transporter

## Abstract

Background: Selenium and zinc are essential trace elements known to regulate cellular processes including redox homeostasis. During inflammation, circulating selenium and zinc concentrations are reduced in parallel, but underlying mechanisms are unknown. Accordingly, we modulated the zinc and selenium supply of HepG2 cells to study their relationship. Methods: HepG2 cells were supplied with selenite in combination with a short- or long-term zinc treatment to investigate intracellular concentrations of selenium and zinc together with biomarkers describing their status. In addition, the activation of the redox-sensitive transcription factor NRF2 was analyzed. Results: Zinc not only increased the nuclear translocation of NRF2 after 2 to 6 h but also enhanced the intracellular selenium content after 72 h, when the cells were exposed to both trace elements. In parallel, the activity and expression of the selenoprotein thioredoxin reductase 1 (TXNRD1) increased, while the gene expression of other selenoproteins remained unaffected or was even downregulated. The zinc effects on the selenium concentration and TXNRD activity were reduced in cells with stable NRF2 knockdown in comparison to control cells. Conclusions: This indicates a functional role of NRF2 in mediating the zinc/selenium crosstalk and provides an explanation for the observed unidirectional behavior of selenium and zinc.

## 1. Introduction

Essential trace elements such as selenium and zinc are micronutrients with indispensable roles in enzymatic reactions, which consequently modify signaling pathways. Aging is accompanied by substantial shifts in trace elements in humans resulting in unidirectional downregulation of selenium and zinc [1]. This downregulation of both trace elements also takes place during acute and chronic inflammation [2]. Another common theme of both trace elements is their suboptimal supply in the European population, which is further aggravated by a diet excluding animal-based foods [3,4].

Selenium exhibits its function by catalyzing redox reactions as part of selenocysteine at the active site of selenoproteins. Those selenoproteins are encoded by 25 genes in humans [5]. Of those, glutathione peroxidases (GPXs) reduce hydroperoxides to their respective alcohols by using glutathione as the reduction equivalent, while thioredoxin reductases (TXNRDs) depend on reduced thioredoxin [6]. During selenium deficiency, the mRNA and protein levels of certain selenoproteins are degraded or decreased, whereas others are maintained [7]. The group of selenium-sensitive selenoproteins comprises, for example, GPX1 or selenoprotein H (SELENOH) [8], while TXNRD1, TXNRD2, or GPX4 belong to the selenium-insensitive selenoproteins maintained under conditions with limited selenium supply [9]. The knowledge about selenium uptake from the intestinal lumen is scarce. But it is well established that the liver is the main organ regulating the systemic selenium homeostasis by expressing and releasing selenoprotein P (SELENOP). SELENOP contains up to 10 selenocysteine residues and can be taken up by target cells via members of the low-density lipoprotein receptor-related protein (LRP) family including LRP1, 2, and 8 (apolipoprotein E receptor 2 (APOER2)) [10,11,12]. In addition, it has been discussed that the cystine/glutamate antiporter solute carrier family 7 member 11 (SLC7A11; also known as XCT) is involved in the uptake of selenite [13,14].

Zinc is bound by approximately 3000 proteins and 300 enzymes [15]. Zinc finger transcription factors form the largest proportion of zinc-binding proteins and coordinate zinc between the sulfur moieties of four cysteine residues or two cysteines and two histidines [16,17]. Zinc can have a catalytic, co-catalytic, or structural role [17]. Because zinc is redox-inactive, the catalytic function of zinc is not mediated by the zinc ion itself, but rather by acting as an electron pair acceptor [18]. To maintain intracellular zinc concentrations, cells use transporters and zinc-binding proteins. The zinc/iron-regulated transporter (Zrt/Irt)-like protein (ZIP) family mediates the import of zinc across the cell membrane on the one hand and the transport from the intracellular compartments into the cytosol on the other hand. The zinc transporter (ZNT) family decreases intracellular zinc concentrations as they transport zinc from the cytosol into the extracellular space or into the lumen of cell organelles [19]. In addition to the ZIP and ZNT proteins, members of the metallothionein (MT) family regulate the intracellular zinc concentration by binding 5–15% of cytosolic zinc [20,21]. One MT protein binds up to seven zinc atoms and is known to transfer zinc to additional zinc-binding proteins, especially under oxidizing intracellular conditions [22]. The expression of MT is enhanced by the metal-sensitive transcription factor (MTF1), which has six zinc finger domains and is activated by zinc binding to a metal-dependent activation domain [23,24,25]. Besides zinc, hydrogen peroxide and heavy metals can indirectly activate MTF1 because they promote the zinc release from proteins such as MT, allowing zinc to bind to MTF1 [26]. Classical MTF1 target genes regulate zinc homeostasis, such as MT1, MT2, ZNT1, and ZNT2 [27,28,29,30].

Besides MTF1, zinc has also been described to activate the transcription factor nuclear factor erythroid 2-related factor 2 (NRF2) [30,31,32]. NRF2 is known as the master regulator of the adaptive response to cellular oxidative stress. Under basal conditions, NRF2 is constantly degraded, which is mediated by its binding to the kelch-like ECH-associated protein 1 (KEAP1), which acts as an adaptor for an ubiquitin ligase. Upon the oxidation of thiol groups of KEAP1, NRF2 is stabilized, and newly synthetized NRF2 can translocate into the nucleus [33,34]. In addition to the classical activation by oxidants and electrophiles, zinc is also able to modulate KEAP1 resulting in NRF2 stabilization and activation [35,36]. There are a plethora of NRF2 target genes acting as antioxidant enzymes, in xenobiotic transformation, iron homeostasis, carbohydrate and lipid metabolism, autophagy, and proteostasis [37]. In addition, two selenoproteins, GPX2 and TXNRD1, have been identified as NRF2 target genes [38,39], but MT1 also has a functional antioxidant-responsive element in the promoter region [40]. Accordingly, we aimed to understand how modulating the selenium and zinc supply of cells in parallel would affect NRF2 and downstream processes. Using HepG2 cells, we showed that TXNRD activity and TXNRD1 expression were highest in cells with both selenium and zinc supply, which was regulated by NRF2. In addition, zinc increased the selenium concentration of cells, which appears to be regulated by transport proteins such as ZIP8, XCT, and APOER2.

## 2. Materials and Methods

### 2.1. Cell Culture and Generation of Stable Knockdown Cells

Cell culture experiments were performed with hepatocarcinoma-derived HepG2 cells derived from a male (ACC 180; German collection of microorganisms and cell cultures). HepG2 cells were further used to generate stable NRF2 knockdown cell clones according to [41], and the previously described stable scramble HepG2 cell line was used as the control. In brief, HepG2 cells were transfected with the plasmid psiRNA-h7SKneo encoding for shRNA against NRF2 with the final sequence 5′-GATGCCACATCAACAC AGAT-3′. Cells were cultured in RPMI medium (Thermo Fisher Scientific (Waltham, MA, USA)) supplemented with 2.5% or 10% fetal calf serum (FCS, Merck KGaA, Darmstadt, Germany), 1% penicillin-streptomycin (Thermo Fisher Scientific), and 1% GlutaMAX™ (Thermo Fisher Scientific) under standard culture conditions (37 °C, 5% CO_2_). Unless otherwise specified, cells were supplied with 50 nM sodium selenite (Thermo Fisher Scientific) or 200 nM selenomethionine (SeMet; Merck KGaA) for 72 h in combination with up to 100 µM zinc sulfate (Merck KGaA) for 2–72 h. As a zinc chelator, 500 µM EDTA (Carl Roth (Karlsruhe, Germany)) was added to the media for 24 h. Cell pellets were frozen in liquid nitrogen and stored at −80 °C.

### 2.2. Preparation of Cell Lysates

Cell pellets were lysed in RIPA buffer (50 mM Tris (PanReac AppliChem (Darmstadt, Germany)), 150 mM NaCl (Carl Roth), 2 mM EDTA (Carl Roth), 0.5% sodium deoxycholate (Merck KGaA), 0.1% SDS (PanReac AppliChem), and 1% NP-40 Alternative (Merck KGaA), pH 7.7 with 0.1% protease inhibitor (Merck KGaA) for 15 min at 4 °C and 1200 rpm using the ThermoMixer^®^ (Eppendorf AG, Hamburg, Germany) for analyzing trace elements and protein expression. To prepare protein lysates for enzyme activity assays, frozen cell pellets were homogenized in Tris buffer (100 mM Tris (Carl Roth), 300 mM KCl (PanReac AppliChem), pH 7.6 with 0.1% Triton X-100 (Serva, Heidelberg, Germany), and 0.1% protease inhibitor (Merck KGaA)) using a TissueLyser II (Qiagen, Hilden, Germany) by two homogenizing steps for 30 s at maximum speed. Cellular debris for both types of homogenization was removed by centrifugation (14,000× *g*, 10 min, 4 °C). To generate nuclear fractions, cells were scraped from the plate with lysis buffer I (10 mM HEPES (Carl Roth), 1.5 mM MgCl_2_ (Carl Roth), 10 mM KCl (PanReac AppliChem), 0.5 mM DTT (Merck KGaA), 0.5 mM PMSF (Carl Roth), and 0.1% NP-40 Alternative (Merck KGaA), pH 7.9). After incubation for 7 min at 4 °C under shaking, cells were centrifuged for 1 min at 4 °C and 6800× *g*. Thereafter, NaCl (5 M, Carl Roth) was added to the lysis buffer II (40 mM HEPES (Carl Roth), 400 mM KCl (PanReac AppliChem), 10% glycerol (Carl Roth), 1 mM DTT (Merck KGaA), and 0.1 mM PMSF (Carl Roth), pH 7.9) and the cell pellet was lysed by ultrasonic treatment (10×, 80% amplitude, 0.5 s cycle), and centrifuged for 30 min at 4 °C and 20,000× *g* to obtain nuclear lysates. Protein concentrations of lysates were determined by Bradford analysis (Bio-Rad Laboratories, Munich, Germany).

### 2.3. Cell Viability Assay

Cells were detached by trypsin/EDTA (ThermoFisher Scientific) and washed with phosphate-buffered saline (140 mM NaCl (pH 7.4 (Carl Roth)), 10 mM Na_2_HPO_4_ (Carl Roth), 2.99 mM KH_2_PO_4_ (Carl Roth)). The cell suspension was used to determine the number of viable and dead cells after staining with 0.4% trypan blue (Merck KGaA) using a Vi-Cell XR Cell Viability Analyzer (Beckman Coulter, Brea, CA, USA).

### 2.4. Measurement of Trace Element Concentrations

The concentrations of zinc, copper, and selenium were measured in RIPA cell lysates using a bench-top total reflection X-ray fluorescence (TXRF) spectrometer (S2 Picofox™ (Bruker Nano GmbH, Berlin, Germany). As an internal standard, 1 mg/L Yttrium (Merck KGaA) was used. Next, 10 µL of each sample were placed on siliconized quartz glass carriers and dried at 40 °C. Samples were measured in triplicate for 1000 s. Trace element concentrations were normalized to the protein content of the sample.

### 2.5. Enzyme Activities

Measurements of NAD(P)H:quinone oxidoreductase (NQO1) [42], TXNRD [43], and GPX [44] activities have been described previously. Briefly, NQO1 activity was examined by a menadione-mediated reduction of 3-(4,5-dimethylthiazol-2-yl)-2,5-diphenyltetrazolium bromide (MTT (Merck KGaA)). TXNRD activity was measured by the NADPH-dependent reduction of 5,5′-dithiobis (2-nitrobenzoic acid) (DTNB (PanReac AppliChem)). GPX activity was determined in a NADPH-consuming glutathione reductase coupled assay. All measurements were performed in triplicate using 96-well plates and a microplate reader (Synergy H1 (BioTek, Bad Friedrichshall, Germany)) and were normalized to protein concentration.

### 2.6. Western Blot

Protein lysates were mixed with loading buffer (41.7 mM Tris pH 6.8 (PanReac AppliChem), 10% glycerin (Carl Roth), 2% SDS (PanReac AppliChem), 0.125% bromophenol blue (Carl Roth), and 2.5% β-mercaptoethanol (Merck KGaA)) and heated for 5 min at 95 °C. Proteins were separated on SDS polyacrylamide gels (10–15%) followed by immunoblotting on nitrocellulose membranes (Merck KGaA). After immunoblotting, membranes were gently shaken for 1 min in Ponceau-S solution (0.2% Ponceau S (Carl Roth) with 3% trichloroacetic acid (Carl Roth)) and bands were recorded by the ChemiDoc™ MP Imaging System (Bio-Rad). Subsequently, membranes were blocked in 5% non-fat dry milk in Tris-buffered saline (5 mM Tris (pH 7.4 (PanReac AppliChem), 15 mM NaCl (Carl Roth)) containing 0.1% Tween 20 (T-TBS (Carl Roth)) for 1 h at room temperature. The membranes were incubated with the primary antibodies (Table 1) overnight at 4 °C. As a secondary antibody, HRP-conjugated goat anti-rabbit IgG (7074S (Cell Signaling, Denvers, MA, USA, 1:50,000) was incubated for 1 h in 5% non-fat dry milk in T-TBS at room temperature. Proteins were detected using SuperSignal™ West Dura (Thermo Fisher Scientific) and band intensities were quantified densitometrically by the ChemiDoc™ MP Imaging System (Bio-Rad). Protein expression was normalized to Ponceau staining.

### 2.7. Quantitative Real-Time PCR

According to the manufacturer’s protocol, the total mRNA of HepG2 cells was isolated using the Dynabeads™ mRNA DIRECT™ Purification Kit (Thermo Fisher Scientific). The SensiFAST™ cDNA Synthesis Kit (Bioline Meridian Bioscience, Cincinnati, Ohio, USA) was used for cDNA transcription. For real-time PCR, cDNA was combined with 1× PerfeCTa SYBR Green Supermix (Quanta, BioSciences, Beverly, MA, USA), forward and reverse primer (each 250 nM final concentration) in a 96-well plate with a total volume of 10 µL as previously described [46]. Primer sequences are given in Table 2. PCR was performed on a real-time PCR system (MX3005P (Agilent, Santa Clara, CA, USA)) with a heat cycle to 95 °C for 3 min, 40 cycles of denaturation at 95 °C for 15 s, annealing at 60 °C for 20 s, and elongation at 72 °C for 30 s. Standard curves from diluted PCR products were used for relative quantification and all samples and standards were measured in triplicate. For normalization, sample values were normalized to a composite factor based on the reference genes HPRT, RPL13a, and GAPDH.

### 2.8. Statistics

Data are given as means + SD of independent experiments. The statistical analysis was performed with GraphPad Prism 8 Software (San Diego, CA, USA) using a two-way analysis of variance (ANOVA) with Bonferroni’s post-test. A *p*-value below 0.05 was considered statistically significant.

## 3. Results

### 3.1. Zinc Increased the Selenium Concentration of Cells

**Figure 1:**
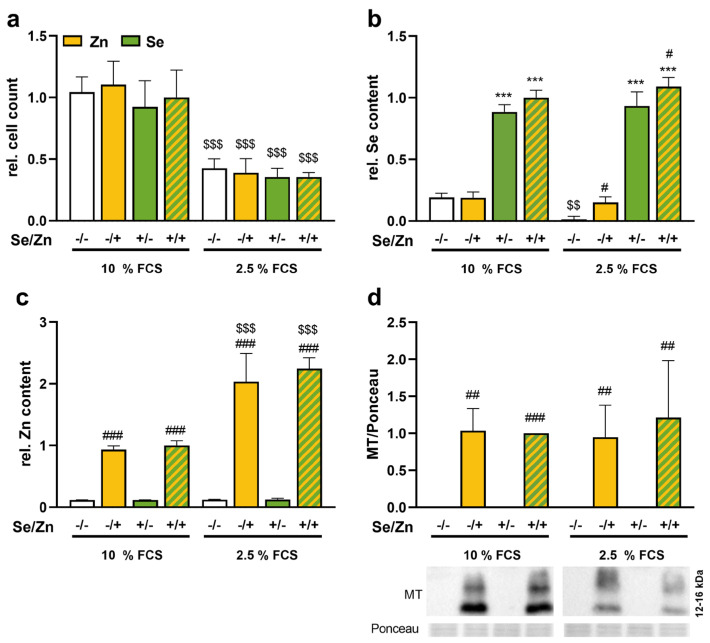
Establishment of culture conditions with 2.5% FBS. HepG2 cells were treated with or without 50 nM sodium selenite in combination with or without 100 μM zinc sulfate for 72 h in media containing 10% or 2.5% FCS. The relative cell count (**a**) was determined by trypan blue exclusion test. Intracellular trace element concentrations of Se (**b**), and Zn (**c**) were determined by total reflection X-ray fluorescence spectrometry (TXRF). The measurement was performed for 1000 s with 1 mg/L yttrium as an internal standard. Protein expression of MT (**d**) was determined by Western blot, normalized to Ponceau staining. Either the −Se/−Zn 10% FBS group (**a**) or the +Se/+Zn group 10% FBS (**b**–**d**) were set as 1. Results are presented as mean + SD (n = 3–4). *** *p* < 0.001 vs. −Se, # *p* < 0.05, ## *p* < 0.01, ### *p* < 0.001 vs. −Zn, $$ *p* < 0.01, $$$ *p* < 0.001 vs. 10% FBS, calculated by two-factorial ANOVA with Bonferroni’s post-test.

In cell culture experiments, FCS is the main source of trace elements, which is standardly added to the media at a level of 10%. Our media with 10% FCS contained concentrations of 5 nM selenium and 2.9 µM zinc. Accordingly, we used media with 2.5% FCS to reduce the basal selenium and zinc concentrations to one fourth. As lower FCS levels potentially reduce cell proliferation, we first counted cells after 72 h of culture. As expected, the number of cells was reduced to about 50% when cells were cultured in media containing 2.5% FCS compared to media containing 10% FCS (Figure 1a). Neither a treatment with 50 nM selenium, 100 µM zinc, nor a combined treatment affected the cell number after 72 h, indicating that there were no cytotoxic effects of the trace elements independently of the FCS concentration used.

The reduction of the FCS concentration to 2.5% indeed reduced the basal selenium concentration drastically compared to cells which were cultured in media with 10% FCS (Figure 1b), while the basal zinc concentration was not affected by the amount of FCS in the media (Figure 1c). Treating the cells with 50 nM selenite resulted in a comparable increase in intracellular selenium concentrations independently of the amount of FCS concentration (Figure 1b). In contrast, zinc administration had a two-times higher effect on intracellular zinc concentrations of cells cultured with 2.5% FCS than of cells with 10% FCS (Figure 1c). The expression of the zinc-sensitive protein MT was only detectable in zinc-treated cells and was neither affected by FCS nor by selenium (Figure 1d). While selenium had no effect on the cellular zinc concentration, zinc increased the cellular selenium concentration but only under the conditions of 2.5% FCS. This effect was independent of the selenocompound used, as it was also observed in cells treated with organic selenomethionine instead of inorganic selenite (Figure S1a). In addition, the zinc chelator EDTA reduced the cellular zinc concentration and concomitantly reduced the selenium concentration in cells treated with zinc and selenite in parallel (Figure S1c,d). For further experiments, only culture conditions with 2.5% FCS were used.

### 3.2. Thioredoxin Reductase 1 Expression Was Increased upon Zinc Treatment

**Figure 2:**
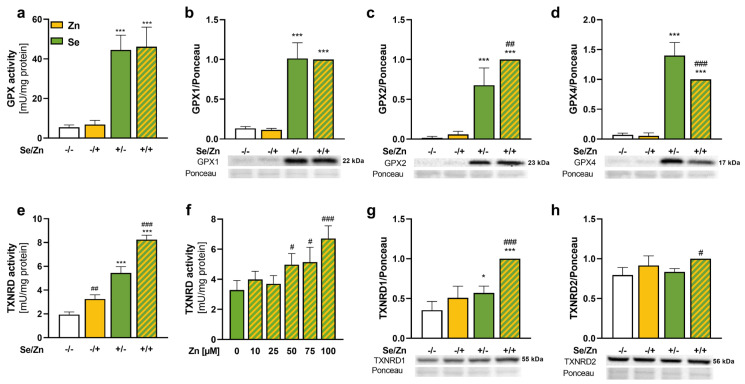
Zinc increased the activity of TXNRD but not of GPX. HepG2 cells were treated with or without 50 nM sodium selenite in combination with or without 100 µM zinc sulfate for up to 72 h (**a**–**e**,**g**,**h**) or with indicated Zn concentrations (**f**) in media containing 2.5% FCS. Enzyme activities of GPX (a) and TXNRD (**e**,**f**) and the protein expression levels of GPX1 (**b**), GPX2 (**c**), GPX4 (**d**), TXNRD1 (**g**), and TXNRD2 (**h**) were determined photometrically (**a**,**e**,**f**) or by Western blot (**b**–**d**,**g**,**h**). Protein expression was normalized to Ponceau staining and presented relative to +Se/+Zn treatment. Results are presented as mean + SD (n = 3–4). * *p* < 0.05, *** *p* < 0.001 vs. −Se, # *p* < 0.05, ## *p* < 0.01, ### *p* < 0.001 vs. −Zn, calculated by two-factorial ANOVA (**a**–**e**,**g**,**h**) or with one-factorial ANOVA (**f**) with Bonferroni’s post-test.

As we observed higher selenium concentrations in HepG2 cells treated with zinc, expression and activity of the selenoprotein families GPX and TXNRD were analyzed. Total GPX activity was only enhanced by selenium but not affected by zinc (Figure 2a). In line with this, the protein expression levels of the isoforms GPX1, GPX2, and GPX4 were at very low levels without selenium treatment and upregulated by selenium (Figure 2b–d). A combined selenium and zinc treatment slightly increased GPX2 expression (Figure 2c), while GPX4 was decreased by zinc (Figure 2d).

Both selenite and selenomethionine were also able to increase TXNRD activity (Figure 2e and S1b). In contrast to GPX activity, the TXNRD activity was significantly increased by zinc both under basal and selenium-treated conditions (Figure 2e and S1b). This effect could further be confirmed by using zinc concentrations ranging from 10 to 100 µM showing a concentration-dependent effect starting from 50 µM zinc (Figure 2f). Selenium treatment increased TXNRD activity by a factor of 2.5, while zinc treatment increased the activity under selenium-deficient and selenium-adequate conditions by 1.7- and 1.5-fold, respectively (Figure 2e). The TXNRD enzyme activity reflects the total activity of cytosolic TXNRD1 and mitochondrial TXNRD2. The TXNRD1 protein expression was highest in cells with combined selenium and zinc treatment (Figure 2g), while the TXNRD2 expression was only mildly enhanced by zinc in combination with selenium (Figure 2h).

### 3.3. Zinc Activated the Transcription Factors NRF2 and MTF1 and Enhanced the mRNA Expression of Their Target Genes

**Figure 3:**
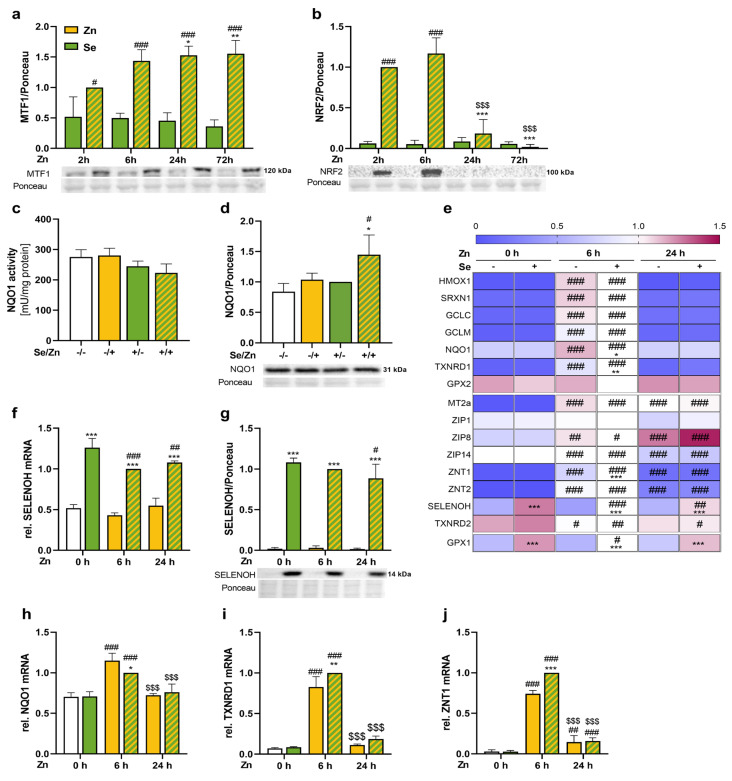
Zinc enhanced the nuclear translocation of NRF2 and MTF1 and increased the expression of their target genes. HepG2 cells were treated with or without 50 nM sodium selenite in combination with or without 100 μM zinc sulfate up to 72 h (**c**,**d**) or treated with selenite for 72 h in combination with or without zinc for the time as indicated (**a**,**b**,**e**–**j**) in media containing 2.5% FCS. Nuclear protein levels of MTF1 (**a**), NRF2 (**b**) and SELENOH (**g**), enzyme activity of NQO1 (**c**), and protein expression of NQO1 (**d**) were determined photometrically (**c**) or by Western blot (**a**,**b**,**d**,**g**). Protein expression was normalized to Ponceau staining and presented relative to samples with selenium treatment in combination with 2 h zinc treatment (**a**,**b**) or to samples with selenium treatment in combination with 6 h zinc treatment (**g**) or samples with selenium treatment (**d**). The mRNA expression levels of NRF2 and MTF1 target genes (**e**–**f**,**h**–**j**) were analyzed by qPCR. Gene expression was normalized to the normalization factor of the reference genes HPRT, RPL13a, and GAPDH and presented relative to samples with selenium treatment and 6 h zinc treatment. Results are presented as mean + SD (n = 3–4). * *p* < 0.05, ** *p* < 0.01, *** *p* < 0.001 vs. −Se, # *p* < 0.05, ## *p* < 0.01, ### *p* < 0.001 vs. −Zn, $$$ *p* < 0.001 vs. 6 h Zn, calculated by two-factorial ANOVA with Bonferroni’s post-test.

Based on the enhanced protein expression of TXNRD1, GPX2, and MT in response to zinc, we aimed to study the activation of relevant transcription factors. As expected, nuclear MTF1 concentrations increased in zinc-treated cells after 6 h and high levels were maintained for up to 72 h (Figure 3a). The nuclear translocation of NRF2 was strongly increased after 2 and 6 h of zinc treatment compared to cells without zinc treatment (Figure 3b). After 24 h of zinc treatment, the nuclear NRF2 concentration reached basal levels again. To analyze NRF2 target genes, NQO1 activity (Figure 3c) and protein expression (Figure 3d) were measured after 72 h of zinc and/or selenium treatment. NQO1 activity was not modulated by any of the treatments. However, NQO1 protein expression was the highest in cells treated with selenium and zinc, as observed for TXNRD1.

In line with the nuclear translocation of NRF2, the mRNA expression of most NRF2 target genes analyzed (hemoxygenase 1 (HMOX1), sulfiredoxin 1 (SRXN1), glutamate-cysteine ligase catalytic subunit (GCLC), glutamate-cysteine ligase regulatory subunit (GCLM), NQO1, TXNRD1) was increased after 6 h of zinc treatment, but counter regulated after 24 h, except for GPX2 which was not modulated by zinc (Figure 3e). The mRNA expression of zinc importers showed a diverse picture in response to zinc. While ZIP1 was not affected, ZIP8 was increased and ZIP14 decreased after 6 and 24 h of zinc treatment (Figure 3e). Interestingly, ZNT1 and 2 showed the same expression pattern as NRF2 target genes, being highest after 6 h of zinc treatment (Figure 3e), even though both of them are described to be MTF1 and not NRF2 target genes [19,29,30]. In contrast, another MTF1 target gene MT2a showed the expression pattern expected from the nuclear levels with highly increased concentrations both after 6 and 24 h (Figure 3e). TXNRD2 which has been described to be downregulated by MTF1 was reduced by zinc treatment both after 6 and 24 h (Figure 3e).

As expected, 72 h selenium pre-treatment increased selenium-sensitive GPX1 mRNA levels independently of the zinc treatment (Figure 3e). Selenium only very moderately affected the mRNA levels of the NRF2 and MTF1 target genes except for SELENOH, which was most responsive to selenium and in parallel was downregulated by zinc (Figure 3f). A combined treatment of zinc and selenium also decreased the protein content of SELENOH in the nucleus after 24 h (Figure 3g). Besides this, selenium effects were only detectable upon a 6 h zinc co-treatment for NQO1 (downregulated by selenium), TXNRD1, and ZNT1 (both upregulated by selenium) (Figure 3h–j).

### 3.4. Zinc-Induced Effects on TXNRD1 and the Selenium Content Were Mediated by NRF2

**Figure 4:**
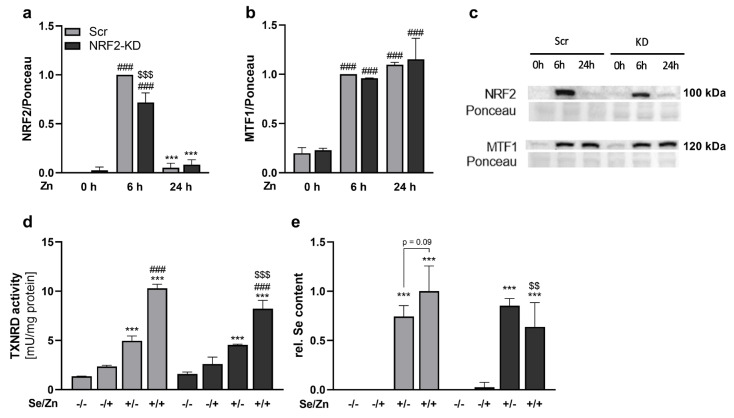
NRF2 partially mediated the zinc effects on selenium homeostasis. HepG2 cells with a stable small hairpin RNA-mediated NRF2 knockdown (NRF2-KD) and scramble (Scr) control cells were treated with 50 nM sodium selenite for 72 h and 100 μM zinc sulfate for the indicated time (**a**–**c**) or for 72 h (**d**,**e**) in media containing 2.5% FCS. The nuclear protein levels of NRF2 (**a**,**c**) and MTF1 (**b**,**c**) were analyzed by Western blot. Protein expression was normalized to the Ponceau staining. Enzyme activities of TNXRD (**d**) and intracellular Se concentrations (**e**) were determined photometrically or by total reflection X-ray fluorescence spectrometry, respectively. The measurement was performed for 1000 s with 1 mg/L yttrium as an internal standard. The results are presented as mean + SD (n = 3). ### *p* < 0.001 vs. –Zn, *** *p* < 0.001 vs. 6 h Zn, $$$ *p* < 0.001 vs. Scr (**a**,**b**) or *** *p* < 0.001 vs. –Se, ### *p* < 0.001 vs. –Zn, $$ *p* < 0.01, $$$ *p* < 0.001 vs. Scr (**d**,**e**) calculated by two-factorial ANOVA with Bonferroni’s post-test.

As zinc strongly activated the NRF2 pathway, we aimed to study its effects in HepG2 cells with stable knockdown of NRF2. Also, in NRF2 knockdown cells, NRF2 translocated into the nucleus in response to zinc after 6 h of treatment but lower nuclear NRF2 concentrations (reduction of 30%) were detectable in comparison to the scramble control cells (Figure 4a). While MTF1 was increased in the nucleus after 6 and 24 h of zinc treatment, it was not affected by NRF2 knockdown (Figure 4b). The zinc-mediated increase in TXNRD activity was also observed in NRF2 knockdown cells but was less pronounced than in scramble cells (Figure 4d). In NRF2 knockdown cells, the selenium concentration was not increased but even reduced by zinc (Figure 4e).

### 3.5. Trace Element Transport Proteins Were Increased by Zinc

**Figure 5:**
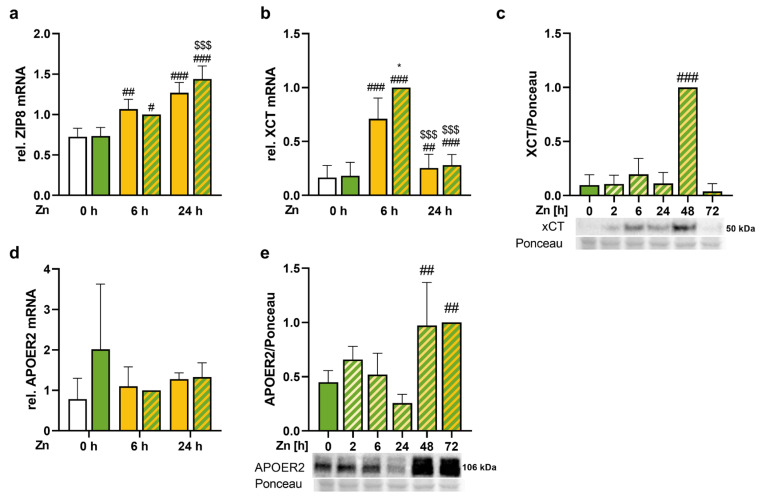
Trace element transporters were increased by zinc. HepG2 cells were treated with or without 50 nM sodium selenite for 72 h in combination with or without 100 μM zinc sulfate for indicated time (up to 72 h) in media containing 2.5% FCS. The mRNA expression levels of ZIP8 (**a**), XCT (**b**), and APOER2 (**d**) were analyzed by qPCR. Gene expression was normalized to the normalization factor of the reference genes HPRT, RPL13a, and GAPDH and was presented relative to samples with selenium and 6 h zinc treatment. XCT (**c**) and APOER2 (**e**) were analyzed by Western blot. Protein expression was normalized to Ponceau staining and presented relative to samples with selenium and 48 h (**c**) or 72 h zinc treatment (**e**). Results are presented as mean + SD (n = 3–4). * *p* < 0.05 vs. −Se; # *p* < 0.05, ## *p* < 0.01, ### *p* < 0.001 vs. −Zn, $$$ *p* < 0.001 vs. 6 h Zn, calculated by two-factorial ANOVA (**a**,**b**,**d**) or with one-factorial ANOVA (**c**,**e**) with Bonferroni’s post-test.

To understand how zinc treatment modulated the selenium concentration of cells, the expression of zinc and selenium transport proteins was analyzed. The mRNA expression of the zinc importer ZIP8, which is also described to import selenium, was increased by a 6 and 24 h treatment with zinc (Figure 5a). In addition, we analyzed the expression of XCT, which has been described to modulate selenite uptake into cells [13]. Also, XCT mRNA expression was increased in response to the short-term zinc treatment, and selenium further increased the gene expression of XCT when treated simultaneously with zinc for 6 h (Figure 5b). This was followed by increased protein expression of XCT after 48 h zinc treatment (Figure 5c). The receptor for SELENOP, APOER2, was not modulated by zinc and selenium on mRNA level (Figure 5d) but showed an increase in protein expression 48 and 72 h after zinc treatment (Figure 5e).

## 4. Discussion

The transcription factor NRF2 is a master regulator of cellular redox balance. The impairment of this balance leads to oxidative stress, a common alteration occurring in many human acute and chronic inflammatory diseases, including cancer, neurodegeneration, metabolic disorders, and aging. Several NRF2 target genes that have been identified belong to the group of antioxidant proteins, the phase II response, metabolic pathways, inflammatory mediators, and trace element homeostasis [33,34]. Thus, many of the liver’s core tasks depend on NRF2. KEAP1 is the main regulator of NRF2 acting as a sensor for electrophiles and other thiol modifying agents. Accordingly, NRF2 inducers can be divided into five categories based on their preferences for specific cysteine residues in KEAP1, which upon modification allow for the nuclear translocation of NRF2 [47].

The two essential trace elements selenium and zinc have been shown to modulate NRF2 activity both in an indirect and direct manner, respectively. Zinc has no direct redox modulating ability because the bivalent cation zinc does not change its oxidation state in biological systems in contrast to iron or copper. But zinc can directly interact with thiol residues, which is vital when binding to the zinc buffering protein metallothionein, which modulates the amount of cellular free zinc [48]. Also, KEAP1 has been reported to be a zinc-thiol protein [49], and therefore, the four cysteine residues that comprise the H_2_O_2_ sensor in KEAP1 may form a similar redox active coordination environment to metallothionein [36]. In addition, zinc is an important component of superoxide dismutase 1 and, thus, also indirectly shapes the cellular redox environment [50]. We have shown before that a short-term treatment of HepG2 cells with 50 µM zinc for 2–6 h results in the nuclear translocation of NRF2, which was again back to basal levels after 24 h [51]. Herein, we used selenium-treated HepG2 cells but again observed the zinc effect on the nuclear translocation of NRF2 (Figure 3b). In addition, classical NRF2 target genes such as HMOX1, NQO1, SRXN1, and GCLC/M were significantly upregulated after 6 h of zinc treatment while again reaching basal levels after 24 h (Figure 3e).

Besides zinc, selenium can also modulate the NRF2 response substantially depending on the concentration applied. While the first reports indicated that high doses of selenite shape a pro-oxidant environment [52], selenium deficiency also resulted in the activation of the NRF2 response [53]. Selenium deficiency is accompanied by the downregulation of antioxidant selenoproteins such as GPXs and TXNRDs as also shown herein both on the protein and the activity level (Figure 2). The activation of NRF2 upon selenium deficiency is discussed to compensate for the loss of antioxidant selenoproteins by upregulating selenium-independent antioxidant proteins such as NQO1, HMOX1, and glutathione transferases [53]. Interestingly, the knockout of selenocysteine tRNA led to an increase in NRF2 target genes such as HMOX1 and SRXN1 [54]. Subsequently, it could be shown in mice that the liver-specific knockout of TXNRD1 and GPX4 activates NRF2 [55,56]. However, even though GPX4 and TXNRD1 expression were both decreased after 72 h of selenium deficiency, there was no increase in NQO1 activity or protein under these conditions (Figure 3c,d). Also, other studies show that the effect of selenium on the NRF2 response is limited in HepG2 cells [57] which might depend on the fact that this is a cancer cell line with impaired NRF2 response.

Next, we aimed to study the interaction of both trace elements selenium and zinc. Unexpectedly, the higher intracellular zinc concentrations in cells with 2.5% FCS resulted in a concomitant increase in the selenium concentrations of the cells both under selenium deficient and adequate conditions (Figure 1). We could also show that this effect was independent of the selenium source as the increase in selenium content was also seen with the organic selenium compound selenomethionine (Figure S1a), indicating that not a direct interaction of zinc and selenite was driving the effect. We used a fourfold higher concentration of selenomethionine than of selenite as this has been shown before to be equally effective in raising intracellular selenium concentrations [46]. To further show the zinc specificity of the observed effect, the zinc chelator EDTA was used, which was able to reduce both the cellular zinc and selenium concentration (Figure S1b,c). To the best of our knowledge, such an effect of zinc on the cellular selenium concentration has not been shown before. Accordingly, we wanted to know whether the higher selenium concentration was used for selenoprotein synthesis. Interestingly, there was no zinc effect on total GPX activity (Figure 2a) but looking at individual isoenzymes of the family revealed an upregulation of GPX2, while GPX4 was downregulated by zinc (Figure 2c,d). GPX2 is a known NRF2 target gene [38], which could explain its upregulation by zinc, but this was not observed on mRNA level after 6 and 24 h of zinc treatment (Figure 3e). In addition to GPX2, TXNRD1 is also known to be regulated by NRF2 [39]. TXNRD1 expression and TXNRD activity (Figure 2e–h), as well as TXNRD1 mRNA expression (Figure 3i), were consistently upregulated by zinc treatment. A combination of the two trace elements led to a synergistic increase in TXNRD activity most probably driven by both an increased TXNRD1 transcription via NRF2 activation and higher selenium availability for selenoprotein translation in zinc-treated cells. Thus, TXNRD1 appears to be the selenoprotein most prominently responding to the zinc stimulation and in addition, the surplus of selenium detected under such conditions might be preferentially bound to TXNRD1. Interestingly, using NRF2 knockdown cells impaired the zinc-induced selenium accumulation and at the same time reduced the zinc-induced increase in TXNRD activity (Figure 4). Previously, it has been discussed that TXNRD1 might act as a negative regulator of NRF2 [58]. Also in this experiment, TXNRD1 upregulation in response to zinc might be important for limiting the NRF2 signal, which would be more effective under adequate selenium conditions.

Finally, we aimed to understand how zinc treatment increased the intracellular selenium concentrations by analyzing different transport proteins involved in zinc or selenium transport. Besides the zinc-binding protein MT, the intracellular zinc concentration is regulated by members of the ZIP (zinc importer) and ZNT (zinc exporter) families. The main transcription factor regulating zinc transport proteins in response to zinc stimulation is MTF1 [48]. Zinc treatment increased the nuclear translocation of MTF1 after 2 h and the effect was maintained for 72 h (Figure 3a). Accordingly, the upregulation of MTF1 target genes such as MT2a, ZNT1, and ZNT2 was highest after 6 h, but in contrast to the NRF2 target genes, they stayed upregulated also after 24 h of zinc treatment (Figure 3e and Figure 5d,e), which is consistent with the literature [24,28,29]. A crosstalk of NRF2 and MTF1 has been described before [59], but herein, the nuclear MTF1 concentrations were not affected by the knockdown of NRF2 (Figure 4b), indicating that such an interaction does not appear to be mainly driving the observed effects. Besides the classical MTF1 target genes involved in zinc homeostasis, two selenoproteins have also been described to be regulated by this transcription factor, namely SELENOH and TXNRD2. These two selenoproteins were downregulated by zinc in the literature [60] and also herein showed reduced mRNA expression both after 6 and 24 h of zinc treatment (Figure 3e). But at the protein level, only SELENOH was reduced by 18% after 24 h of zinc treatment (Figure 3g). Thus, the regulation of these selenoproteins via the zinc-induced activation of MTF1 provides another mechanism of interaction of both trace elements.

Regarding the ZIP family, ZIP8 was of major interest because it was increased by zinc treatment for 6 h and 24 h (Figure 5a) and it is known to not only transport zinc but also other divalent metal ions such as manganese and iron as well as selenite. An increased intracellular selenium concentration was detected in cells and mice with increased ZIP8 expression, while the opposite was observed upon knockdown or knockout of ZIP8 [60,61]. Thus, the upregulation of ZIP8 mRNA levels indicates a putative mechanism modulating the zinc-dependent increase in selenium. In addition, we analyzed XCT and APOER2 expression, both of which are reported to be regulated by NRF2 [62,63]. APOER2 modulates the cellular uptake of SELENOP [10], but this has not been described for the liver so far. Besides its function as a transporter for cystine resulting in the upregulation of intracellular glutathione, a transporter function of XCT for selenite has been described for cancer cells [13,14,64], which might be of relevance for the liver as well. XCT was upregulated on mRNA and protein level, and the protein expression was the highest after 48 h of zinc treatment (Figure 5b,c). In contrast, APOER2 was not significantly modulated by selenium or zinc on mRNA level, but zinc had an effect on APOER2 protein levels which were upregulated after 48 and 72 h of zinc treatment (Figure 5d,e). Thus, all three transporters (ZIP8, XCT, and APOER2) might contribute to the zinc-induced increase in intracellular selenium concentrations.

## 5. Conclusions

It is well established that NRF2 is a master regulator of many fundamental processes in the liver. Herein, we showed that the zinc-induced upregulation of the intracellular selenium concentration is mediated by NRF2, and this most probably involves the upregulation of one or several of three transport proteins which have been shown to increase the selenium uptake. Besides this, the surplus of selenium entering the cells appears to be predominantly used for the synthesis of TXNRD1 for which an NRF2-induced upregulation of its transcript levels happens in parallel. As TXNRD1 has been discussed to act as a shut-off signal for NRF2, zinc and selenium might act together in activating and fine-tuning the NRF2 response, respectively.

## Figures and Tables

**Table 1:** Primary antibodies.

Antibody	Company	Dilution in T-TBS
rabbit anti-APOER2	Abcam (108208)	1:500 in 5% non-fat dry milk
rabbit anti-GPX1	Abcam (8850)	1:5000
rabbit anti-GPX2	[45]	1:1000
rabbit anti-GPX4	Abcam (125066)	1:5000
rabbit anti-MT	Abcam (192385)	1:1000
rabbit anti-MTF-1	Novus Biologicals (86380)	1:250
rabbit anti-NQO1	Abcam (34173)	1:4000
rabbit anti-NRF2	Cell Signaling (12721)	1:1000
rabbit anti-SELENOH	Abcam (151023)	1:500
rabbit anti-TXNRD1	Abcam (16840)	1:5000
rabbit anti-TXNRD2	Abcam (180493)	1:1000
rabbit anti-XCT	Cell Signaling (12691)	1:1000 in 5% BSA

**Table 2:** Primer sequences (fwd and rev).

Primer	RefSeq-ID	Sequence (5′ → 3′)
GAPDH	NM_001289746.1	ACTCATGACCACAGTCCATGCC GATGACCTTGCCCACAGCCT
GCLC	NM_001498.3	TGCTGTCTCCAGGTGACATTCCA GGAGATGCAGCACTCAAAGCCA
GCLM	NM_002061.3	GTTGACATGGCCTGTTCAGTCCT CCCAGTAAGGCTGTAAATGCTCCA
GPX1	NM_000581.2	TACTTATCGAGAATGTGGCGTCCC TTGGCGTTCTCCTGATGCCC
GPX2	NM_002083.4	GTGCTGATTGAGAATGTGGC AGGATGCTCGTTCTGCCCA
HMOX1	NM_002133.2	CAACAAAGTGCAAGATTCTGCCC CTACAGCAACTGTCGCCACC
HPRT	NM_000194.2	TGGCGTCGTGATTAGTGATG GGCCTCCCATCTCCTTCAT
MT2A	NM_005953.3	AGGGCTGCATCTGCAAAGGG TAGCAAACGGTCACGGTCAGGG
NQO1	NM_001025434.1	CATCACAGGTAAACTGAAGGACCC CTCTGGAATATCACAAGGTCTGCG
RPL13A	NM_012423.2	AGCCTACAAGAAAGTTTGCCTATCTG TAGTGGATCTTGGCTTTCTCTTTCCT
SELENOH	NM_170746.2	GCTTCCAGTAAAGGTGAACCCGA TCAGGGAATTTGAGTTTGCGTGG
SRXN1	NM_080725.1	CTCAGTGCTCGTTACTTCATGGTC GTTTGGCCCTTCCTCTTCCTCC
TXNRD1	NM_015762.1	GTGTTGTGGGCTTTCACGTACTG TGTTGTGAATACCTCTGCACAGAC
TXNRD2	NM_006440.3	GTTCCCACGACCGTCTTCAC GTGATAGACCTCAACATGCTCCTG
ZIP1	NM_001271958.2	ATGAAGGCTCAGCTTCCCGC AGCCAGGTAGTCAGGCAGCA
ZIP8	NM_001135146.2	ATGGTCAGAATGGTCATACCCAC GCAGGATTTGCATAGCATGTCAC
ZIP14	NM_001128431.2	GGACCTGGACCACATGATTCCT GTAGCGGACACCTTTCAGCCA
ZNT1	NM_021194.3	GACCAGGAGGAGACCAACAC TCACCACTTCTGGGGTTTTC
ZNT2	NM_001004434.3	CCAGAGCAACCATCACTGCCA GGTACCCACCAACGACTTCTCC

## Data Availability

The raw data supporting the conclusions of this article will be made available by the authors on request.

## Supplementary Materials

The following supporting information can be downloaded at: https://www.mdpi.com/article/10.3390/antiox13101211/s1, Figure S1: Selenium content and TXNRD activity are increased by zinc independent of the selenium species.

## Abbreviations

apolipoprotein E receptor 2 (APOER2); cystine/glutamate antiporter solute carrier family 7 member 11 (SLC7A11, XCT); glutamate-cysteine ligase catalytic subunit (GCLC); glutamate-cysteine ligase regulatory subunit (GCLM); glutathione peroxidases (GPXs); glycerinaldehyd 3-phosphate dehydrogenase (GAPDH); hemoxygenase 1 (HMOX1); hypoxanthine-guanine phosphoribosyltransferase (HPRT); kelch-like ECH-associated protein 1 (KEAP1); low-density lipoprotein receptor-related protein (LRP); metallothionein (MT); metal-sensitive transcription factor (MTF1); NAD(P)H:quinone oxidoreductase (NQO1); nuclear factor erythroid 2-related factor 2 (NRF2); ribosomale protein L13a (RPL13A); selenomethionine (SeMet); selenoprotein H (SELENOH); selenoprotein P (SELENOP); sulfiredoxin 1 (SRXN1); thioredoxin reductases (TXNRDs); zinc transporter (ZNT); zinc/iron-regulated transporters (Zrt/Irt)-like protein (ZIP).
