# Supplementary material for: NRF2 and Thioredoxin Reductase 1 as Modulators of Interactions between Zinc and Selenium
Source: Antioxidants (Basel). 2024 Oct 8;13(10):1211. doi: 10.3390/antiox13101211 (PMC11505002; doi:10.3390/antiox13101211)
Supplement: Supplementary file 1 [file antioxidants-13-01211-s001.zip › antioxidants-3230401-supplementary.pdf]

Supplementary

NRF2 and thioredoxin reductase 1 as modulators of interactions between zinc and selenium

Alina Loeser[1,2], Maria Schwarz[1,2], Anna Patricia Kipp[1,2]

[1]Department of Nutritional Physiology, Institute of Nutritional Sciences, Friedrich Schiller University Jena, 07743 Jena, Germany

[2]TraceAge-DFG Research Unit on Interactions of Essential Trace Elements in Healthy and Diseased Elderly, Potsdam-Berlin-Jena-Wuppertal, 14558 Nuthetal, Germany

Address for correspondence:

Department of Nutritional Physiology, Institute of Nutritional Sciences, Friedrich Schiller University Jena, Dornburger Str. 24, 07743 Jena, Germany

Email: anna.kipp@uni-jena.de

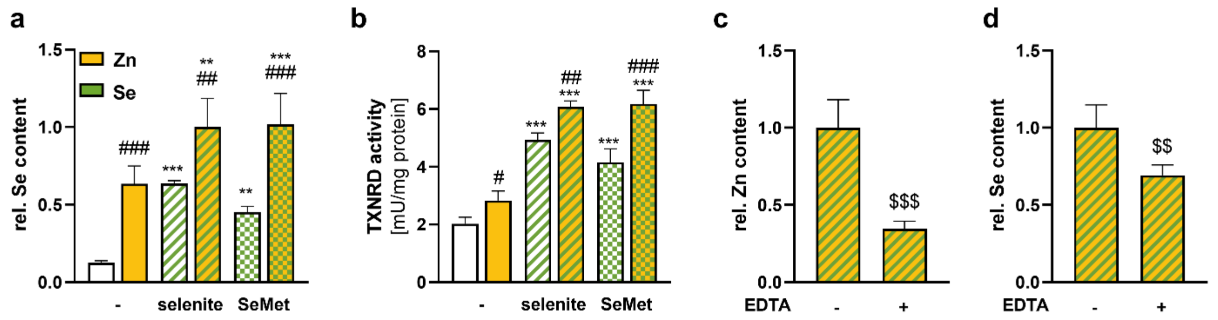

Figure S1: Selenium content and TXNRD activity are increased by zinc independent of the selenium species. HepG2 cells were treated with or without 50 nM sodium selenite or 200 nM selenomethionine in combination with or without 100 µM zinc sulfate for 72 h and cultured in media containing 2.5 % FCS (A and B). HepG2 cells were treated with 50 nM sodium selenite and 100 µM zinc sulfate with or without 500 µM ETDA for 24 h and cultured in media containing 10 % FCS (C and D). Intracellular trace element concentrations of Se (A and D) and Zn (C) were determined by total reflection X-ray fluorescence spectrometry. The measurement was performed for 1000 s with 1 mg/l yttrium as an internal standard. The mean of samples with sodium selenite treatment in combination with zinc treatment or without EDTA were set as 1. Enzyme activity of TXNRD (B) was determined photometrically. Results are presented as mean + SD (n = 4). ***$p < 0.001$ vs. -Se, $^{\#}p < 0.05$, $^{\#\#}p < 0.01$, $^{\#\#\#}p < 0.001$ vs. -Zn, $^{\$\$}p < 0.01$, $^{\$\$\$}p < 0.001$ vs. -EDTA, calculated by two-factorial ANOVA with Bonferroni´s post-test or by t-test.
